# Incremental Value of a Dedicated Head and Neck Acquisition during ^18^F-FDG PET/CT in Patients with Differentiated Thyroid Cancer

**DOI:** 10.1371/journal.pone.0162482

**Published:** 2016-09-06

**Authors:** Renaud Ciappuccini, Nicolas Aide, David Blanchard, Jean-Pierre Rame, Dominique de Raucourt, Jean-Jacques Michels, Emmanuel Babin, Stéphane Bardet

**Affiliations:** 1 Department of Nuclear Medicine and Thyroid Unit, François Baclesse Cancer Centre, Caen, France; 2 INSERM U1086 Cancers & Préventions, Normandie University, Caen, France; 3 INSERM U1199 BioTICLA Unit, François Baclesse Cancer Centre, Caen, France; 4 Normandie University, Caen, France; 5 Department of Nuclear Medicine, University Hospital, Caen, France; 6 Department of Head and Neck Surgery, François Baclesse Cancer Centre, Caen, France; 7 Department of Pathology, François Baclesse Cancer Centre, Caen, France; 8 Department of Head and Neck Surgery, University Hospital, Caen, France; Fu Jen Catholic University, TAIWAN

## Abstract

**Objectives:**

^18^F-FDG-PET/CT is a useful tool used to evidence persistent/recurrent disease (PRD) in patients with differentiated thyroid cancer and iodine-refractory lesions. The aim of this study was to compare the diagnostic value at the cervical level of the routine whole-body (WB) acquisition and that of a complementary head and neck (HN) acquisition, performed successively during the same PET/CT study.

**Methods:**

PET/CT studies combining WB and HN acquisitions performed in 85 consecutive patients were retrospectively reviewed by two nuclear medicine physicians. ^18^F-FDG uptake in cervical lymph nodes (LN) or in the thyroid bed was assessed. Among the 85 patients, the PET/CT results of the 26 who subsequently underwent neck surgery were compared with surgical and pathological reports. The size of each largest nodal metastasis was assessed by a pathologist.

**Results:**

In the 85 patients, inter-observer agreement was excellent for both WB and HN PET/CT interpretation. Of the 26 patients who underwent surgery, 25 had pathology proven PRD in the neck. Of these 25 patients, 15 displayed FDG uptake on either WB or HN PET. In these 15 patients, HN PET detected more malignant lesions than WB PET did (21/27 = 78% vs. 12/27 = 44%, P = 0.006). Node/background ratios were significantly higher on HN than on WB PET (P<0.0001). Three false-negative studies (20%) on WB PET were upstaged as true-positive on HN PET. The mean size of the largest LN metastasis was 3 mm for the LN detected neither on WB nor on HN PET, 7 mm for the metastasis detected on HN but not on WB PET, and 13 mm for those detected on both acquisitions (P = 0.0004). Receiver-Operating Characteristic analysis showed that area under the curve was higher for HN PET than for WB PET (0.97 [95%CI, 0.90–0.99] vs 0.88 [95%CI, 0.78–0.95], P = 0.009).

**Conclusions:**

HN acquisition improves the ability to detect PRD in the neck compared with WB acquisition alone. We recommend systematically adding an HN acquisition when PET/CT is performed to detect PRD in the neck.

## Introduction

Persistent/recurrent disease (PRD) in the neck occurs in 10% to 40% of patients with differentiated thyroid cancer (DTC) [1,2]. When neck lesions are isolated without distant metastases, surgery achieves permanent remission in a significant number of patients [3,4]. Accurate imaging is therefore crucial to optimally plan surgery. In the case of iodine-avid lesions, post-^131^I whole-body (WB) scintigraphy with complementary SPECT/CT images after therapeutic [5] or diagnostic [6] activity well supports the surgeon to find and to remove the malignant lymph nodes (LN), sometimes using radioiodine-guided surgery [7]. 18-Fluorodeoxyglucose positron emission tomography with computed tomography (^18^F-FDG-PET/CT) has also proved to be a relevant functional imaging tool in distant iodine-refractory lesions [8–10] and also in the neck [11]. Few studies have focused on the neck with a complementary acquisition in patients with DTC [12–14] and confirmation with pathology is lacking. Nevertheless, it has been shown in head and neck cancers that a dedicated head and neck PET/CT protocol could improve the detection of small LN metastases in comparison to the WB protocol [15]. Furthermore, recent improvements have been made in PET imaging, such as advanced reconstruction algorithms that model the point spread function (PSF). It is expected that such algorithms will enable more accurate detection of small metastases [16,17].

The aim of the this study was to assess in DTC patients the diagnostic performances at neck level of a dedicated head and neck (HN) acquisition immediately performed after a WB acquisition during the same PET/CT session.

## Materials and Methods

### Patients

From August 2009 to May 2013, 85 consecutive DTC patients with a negative post-therapeutic ^131^I WB scan underwent at least one PET/CT study with both WB and HN acquisition. The first PET/CT study combining both acquisitions was retrospectively reviewed. All patients had high serum thyroglobulin (Tg) levels except one with an increase in the Tg antibodies level. These 85 patients were referred for PET/CT due to biochemical and/or neck US abnormalities (n = 50), follow-up of metastatic disease (n = 28) or assessment before RAI ablation for high risk DTC (n = 7). Of 85 patients, 26 underwent neck (n = 24) or upper thorax (n = 2) surgery.

### Ethics Statements

The study procedures were in accordance with the ethical standards of the committees with responsibility for human experimentation and with the 1964 Helsinki declaration and its later amendments. The study was approved by the local institution review board of François Baclesse Cancer Centre (Basse-Normandie) and the requirement to obtain informed consent was waived. The patient records and information were anonymized and de-identified prior to analysis.

### PET/CT imaging protocol

Calibration of the PET system, cross calibration between the PET system and the dose calibrator, and PET/CT examinations were performed as per the EANM guidelines [18,19]. PET/CT images were acquired using a PET/CT scanner (Biograph 6, Siemens Medical Solutions) containing a six-slice spiral CT component. Patients fasted for at least 6 h before injection of 3.9 ± 0.4 MBq per kg of FDG. Fingerstick glucose was 5.61 ± 1.32 mmol/l. WB PET/CT images started 62 ± 3 min after injection (5 or 6 bed positions depending on the patient’s height; 2 min and 40 s per bed position in patients of low and average weight, or 3 min and 40 s per bed position in overweight patients) from mid-thigh to the base of the skull, with arms elevated when possible. CT scan of the WB PET/CT was acquired with the following parameters: 60 mAs; 130 kV; slice thickness, 3 mm; and pitch, 1. No intravenous contrast agent was administrated. Immediately after completion of WB acquisition and patient repositioning (i.e., 6 ± 5 min after the end of the WB PET images), an HN PET/CT data set was acquired from the base of the skull to the upper thorax, with arms positioned along the body using one bed position of 8 min. CT scan of the HN PET/CT protocol was acquired with the following parameters: 100 mAs; 130 kV; slice thickness, 2.5 mm; and pitch, 1.

All PET data sets were reconstructed with a PSF reconstruction algorithm (HD; TrueX, Siemens Medical Solutions; 3 iterations and 21 subsets), using a 168×168 matrix and a 256×256 matrix for WB PET and HN PET, respectively. No post-filtering was used, as modelling the PSF during iterative reconstruction introduces correlations between neighbouring voxels in a manner similar to smoothing filters and has thus been shown to achieve maximal performance with little or no filtering [20]. Time of PET/CT acquisitions was extracted from Digital Imaging and Communications in Medicine (DICOM) headers.

### PET/CT interpretation

Overall, 170 anonymized PET/CT data sets (85 WB PET/CT images and 85 HN PET/CT images) were reviewed after randomisation (Prism random number generator, GraphPad software) by two board-certified nuclear medicine physicians (S.B. and R.C.) during separate reading sessions on a digital workstation (Siemens Medical Solutions). Each reviewer independently and blindly reviewed the 170 PET/CT studies in a random order. WB and HN PET/CT images were not paired together during interpretation. For each study, the reviewer had only knowledge of the pathology, the side and the TNM classification (7^th^ edition) of the primary thyroid tumor. PET images (with and without attenuation correction), CT images and fused PET/CT images were available for interpretation. WB PET/CT images were reviewed from the base of the skull to the upper thorax region. For visual analysis, each FDG focus on WB or HN PET images was reported and was graded on a 5-point scale (1, definitely benign; 2, probably benign; 3, indeterminate; 4, probably malignant; and 5, definitely malignant). In case of LN uptake, attention was focused to a neck compartment according to the Robbins classification [21]. When present, metastatic FDG lesions in the lungs or bones were not reported. Maximum standardized uptake value (SUVmax) of each focus was measured by drawing a circular ROI of 12 mm [18] on the slice where the pathological foci displayed the highest activity. Mean standardized uptake value (SUVmean) of the background was measured by drawing a circular ROI of 12 mm in the sternocleidomastoid muscle for the muscle background and in the jugulo-carotid vessels for the vascular background, respectively. Node/background (N/B) ratios were computed as the ratio between the SUVmax in the node and the SUVmean in the background (muscle and vascular background, respectively). The presence of lesions on co-registered CT image sets in front of each FDG focus was also reported. When a LN or a mass in the thyroid bed was measurable, its short-axis dimension and shape were reported. CT data were interpreted according to Choi et al. [22], i.e. a LN visible on co-registered CT images was scored benign if it did not have a round shape, its small axis < 10 mm, there was no calcification, and there were no cystic features. Otherwise, it was scored malignant. At patient level, the PET/CT images data set was scored negative if the foci were all scored 1 or 2, indeterminate if no foci scored higher than score 3, and positive if at least one score 4 or 5. Discrepancies were resolved by a consensus review.

### Neck surgery and pathology

The recommendation for surgery was made after local multidisciplinary meetings based on clinical, biological and imaging data, including PET/CT. Twenty patients were operated on in our institution (J.-P.R., D.B. and D.D.R.) and six in other institutions. Surgical and pathological reports were available for all patients. The LN dissection procedure, the number of LN removed, the number of malignant LN and their location were specified. After formalin fixation and paraffin embedding of the entire node, histological slides (5-μm thick) were stained with haematoxylin and eosin for histopathological examination. All pathological specimens were analysed by the same pathologist (J.-J.M.) who measured the largest diameter of each nodal metastatic deposit. The largest tumoral size of each malignant node was measured in all the patients with neck LN involvement except for patient 11. This patient was operated on 2 years after PET/CT when LN involvement had significantly increased during PET/CT follow-up. Surgery and pathology were used as the gold standard.

### Statistical analysis

Quantitative data are presented as mean ± standard deviation (SD) or median (min-max) if necessary. Weighted kappa was used for inter-observer agreement and agreement between PET data sets. The node/background ratios were compared using the non-parametric Mann-Whitney test for paired samples. The sizes of the largest metastasis depending on the status for WB PET and HN PET were compared using the Kruskal–Wallis rank sum test. Statistical significance was defined as P<0.05. Prism (GraphPad software) and Vassar University clinical research calculators were used for graphs and statistics (http://vassarstats.net/). Receiver-Operating Characteristic (ROC) curve analysis was performed with Medcalc (version 11.6.1). The areas under the curve (AUC) were compared using the Hanley test.

## Results

### Inter-observer agreement

In the 85 DTC patients, the inter-observer agreement at patient level (negative, indeterminate, positive) was excellent for both WB PET/CT (weighted kappa = 0.89, CI95%: 0.82–0.96) and HN PET/CT interpretation (weighted kappa = 0.85, CI95%: 0.77–0.93).

**Fig 1:**
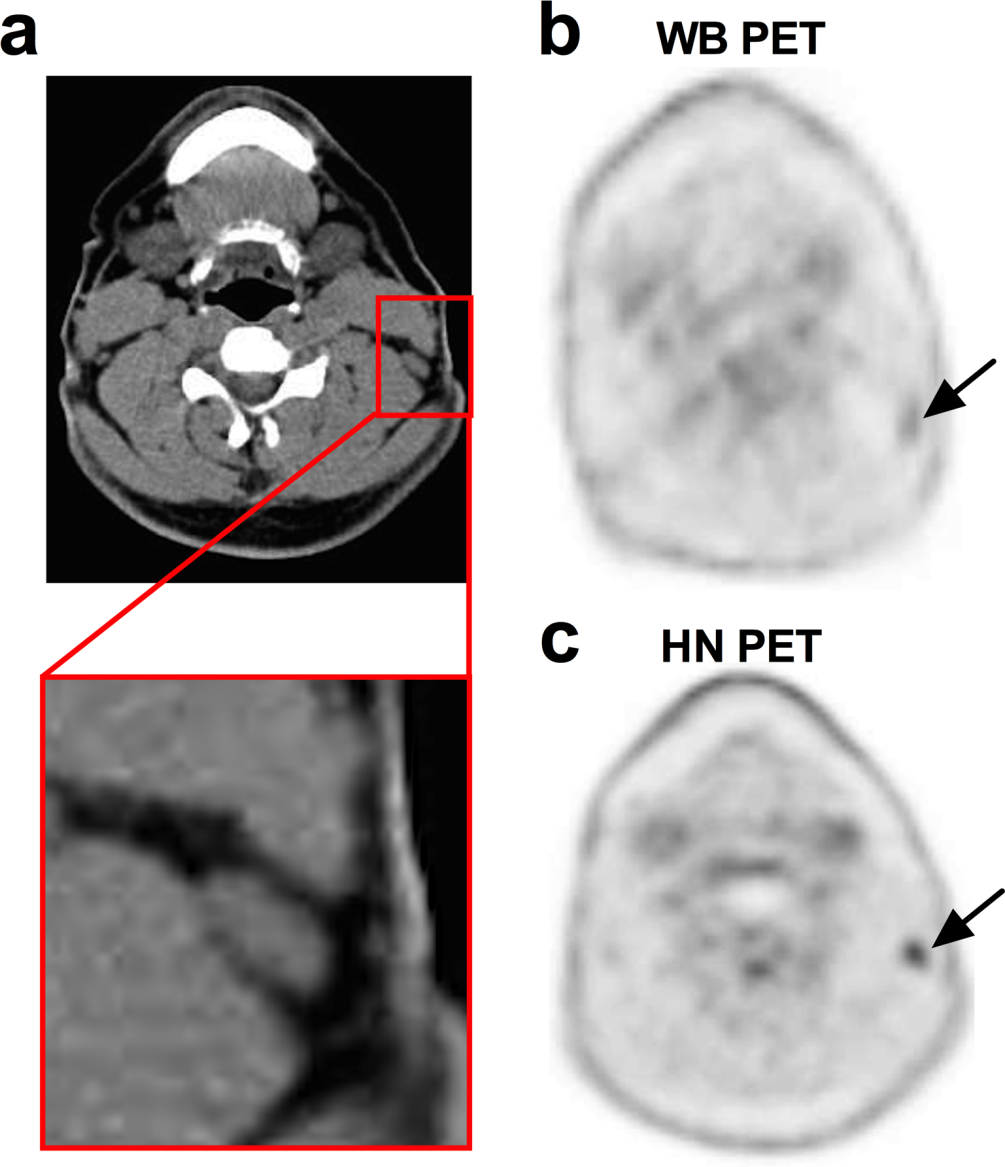
PET/CT study in a 43-yr-old patient with papillary thyroid cancer and central nodal disease. He presented a detectable rhTSH-stimulated serum Tg level (4 ng/ml) three years after initial surgery. The neck US and diagnostic ^131^I whole-body (WB) scan were negative. Hybrid CT scan of the PET/CT study is presented in Panel a. On the WB PET (Panel b) there was a faint FDG uptake (SUVmax = 2.1) in a left lateral LN which was considered as probably benign (score 2) by Reader 1 and indeterminate (score 3) by Reader 2. On head and neck (HN) PET (Panel c), both readers reported a probably malignant (score 4) FDG focus (SUVmax = 3.4). Node/muscle background ratio was 4.3 on HN PET vs. 2.3 on WB PET. A LN without suspicious features (short-axis: 6 mm; long axis: 14 mm) was described in front of this FDG focus on hybrid CT scan (Panel a).

An example of PET/CT with a discrepancy in image analysis by the two readers is presented in Fig 1.

In the 26 DTC patients who underwent surgery, the inter-observer agreement at patient level was also excellent for both WB PET/CT (weighted kappa = 0.92, CI95%: 0.82–1.00) and HN PET/CT interpretation (weighted kappa = 0.83, CI95%: 0.68–0.94).

Of the 59 patients who did not undergo surgery, 21 had neck PRD. These patients were not operated on because of distant metastases or contra-indication to surgery. Twenty patients had FDG foci scoring 4 or 5 in the neck.

### Results in the surgical group

Of the 26 PET/CT studies of patients who underwent surgery, 21 (81%) were performed on levothyroxine treatment and 5 after stimulation, either after recombinant human thyrotropin (rhTSH) (n = 4) or after thyroid hormone withdrawal (n = 1). Recurrence was observed 3.4 ± 2.9 years after initial diagnosis (i.e. thyroidectomy). Surgery was performed 9 ± 7 weeks after PET/CT (except for Patient 11). The surgical procedures consisted of seven central neck dissections, 12 lateral, three central and lateral, and four adenectomies (two in the lateral compartment and two in the upper mediastinum). Twenty-two patients had unilateral and four bilateral neck dissection. Malignant lesions were found in all patients but one. Pathology showed papillary thyroid cancer (72%), poorly differentiated thyroid carcinoma (20%) or Hürthle-cell carcinoma (8%). Of the 25 patients with malignant lesions, 15 had a positive PET/CT with at least one FDG-avid lesion (score ≥ 4). The characteristics of these 15 patients are presented in Table 1. Thirteen patients had pathologically proven LN disease and two had local recurrence in the thyroid bed.

**Table 1:** Characteristics of the 15 patients operated on in the neck with at least one FDG focus scoring 4 or 5 on PET/CT.

	Sex/ Age	TNM 2009 7^th^ ed.	Pathology	Tg on LT4/ stimulated Tg (ng/ml)	PET/CT				Neck US	Surgery/Pathology		Disease status at last visit
					Preparation modality	Number of grade 4–5 foci		Number of abnormal LN on hybrid CT of WB/HN	Number of abnormal LN			
						WB	HN			MalignantLN/total removed	Size^d^ (mm)	
1	M/59	T1aN1bM0	PTC	0.8/5.9	rhTSH	0	1	0/0	1	1/1	13	NERD
2	F/73	T3N1bM0	PDTC	1414/ NP	on LT4	1	1	1/1	1	1/2	21	SD
3	M/62	T3N1bM0	PTC	5.7/NP	on LT4	1	1	0/0^b^	0	1/1	5	SD
4	F/63	T1aN1aM0	PTC	6/162	THW	0	1	0/0^b^	0	1/2	8	BD
5	M/27	T3N1bM0	PDTC	21/NP	on LT4	2	2	0/1^b^	1	2/4	12	BD
6	F/65	T2N1aM0	PTC	2.9^a^/NP	on LT4	2	3	0/0	2	6/45	6	BD
7	M/63	T2N0M0	PDTC	9.3/NP	on LT4	1	2	0/0	0	2/2	5	Dead-PD
8	M/76	T3N1aM0	PTC	1.2/6.8	rhTSH	1	1	1/1	NP	2/21	9	SD
9	M/65	T4aNxM1	PDTC	14408/NP	on LT4	4	7	2/2^b^	4	LR	NA	Dead-PD
10	F/21	T3N1bM0	PTC	1/NP	on LT4	1	3	0/0	1^c^	5/31	5	NERD
11	F/74	T4aN1bM1	PTC	14/NP	on LT4	1	2	0/0	NP	2/4	NA	SD
12	F/63	T2N1aM0	HCC	188/NP	on LT4	3	5	1/2	NP	LR	NA	SD
13	M/40	T3NxM0	PDTC	29/NP	on LT4	1	2	0/0^b^	NP	2/2	6	SD
14	F/42	T3N1bM0	PTC	14/NP	on LT4	0	1	0/0	1	1/26	4	NERD
15	M/71	T3N1aM0	HCC	10/NP	on LT4	1	1	0	0	1/4	7	BD

^a^ Presence of AntiTg antibodies

^b^ a diagnostic CT scan with contrast media injection was performed and reported as normal

^c^ false positive

^d^ Size: refers to the tumoral size of the smallest node evidenced on PET/CT and measured by the pathologist; PTC: papillary thyroid carcinoma; PDTC: poorly differentiated thyroid carcinoma; HCC: Hürthle cell carcinoma; Tg: thyroglobulin; LT4: levothyroxine; rhTSH: recombinant human TSH (Thyrogen®); THW: thyroid hormone withdrawal; NP: not performed; WB: whole-body acquisition; HN: dedicated head and neck acquisition; LR: local recurrence without node involvement; NA: not applicable; NERD: no evidence of residual disease; SD: structural disease; BD: biochemical disease; Dead-PD: died from DTC-progressive disease.

#### PET/CT interpretation at patient level

**Fig 2:**
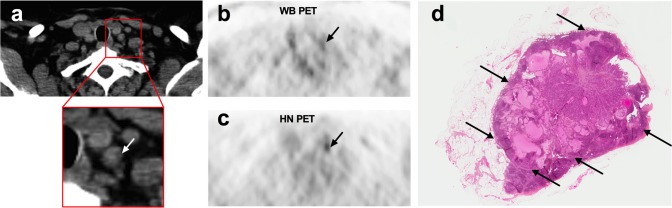
PET/CT study in a 63-yr-old patient (Patient 4) with a pT1aN1aM0 papillary thyroid cancer and a detectable serum Tg level under levothyroxine (6 ng/ml). Hybrid CT scan of the PET/CT study is presented in Panel a. Whole-body (WB) PET (Panel b) was considered as normal whereas a small grade 4 FDG focus was reported on head and neck (HN) PET (Panel c) by both readers. Node/muscle background ratio was 3.9 on HN PET vs. 1.9 on WB PET. A small LN without suspicious features (short-axis: 7 mm; long axis: 9 mm) was described in front of this FDG focus (Panel a). Neck US examination was normal. Central neck lymph node dissection was performed and pathology confirmed HN PET/CT data. The tumor size of the left central LN was 8 mm at histology (Panel d).

Of 26 patients, 15 were true-positive (score ≥ 4) on HN PET and 12 on WB PET. Three patients (patients 1, 4 and 14) were therefore correctly upstaged as malignant on HN PET as illustrated in Fig 2. One patient was truly negative on both PET acquisitions. Ten patients were falsely-negative (score ≤ 3) on both PET acquisitions.

#### PET/CT interpretation at nodal level

**Fig 3:**
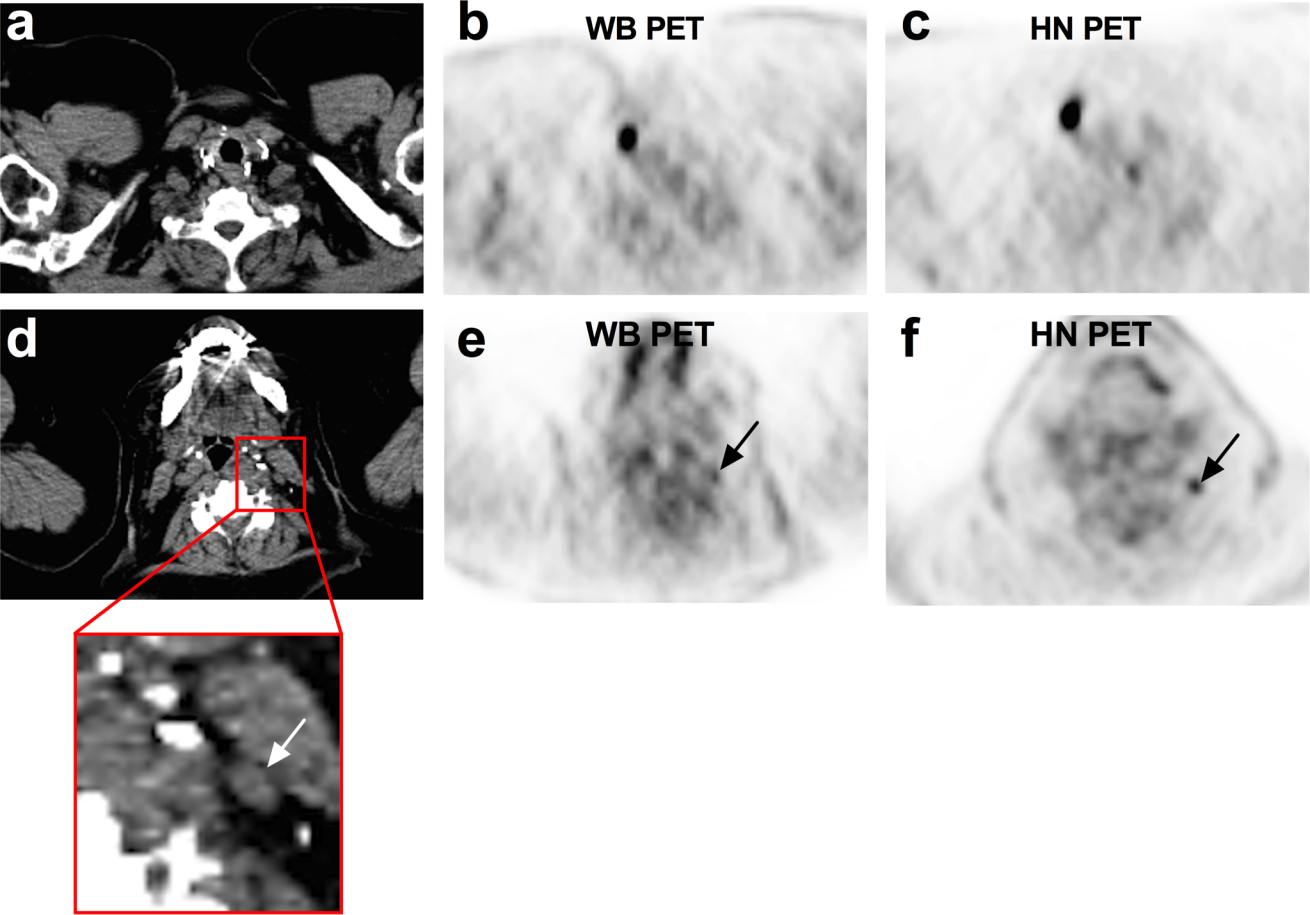
PET/CT study in a 74-yr-old patient (Patient 11) with a pT4aN1bM1 papillary thyroid cancer (tall-cell variant). She presented with a detectable serum Tg level under levothyroxine (14 ng/ml) 5 years after initial diagnosis. Both whole-body (WB) and head and neck (HN) PET (Panels b and c) evidenced a grade 5 FDG focus in front of an abnormal LN (short-axis: 10 mm; long axis: 16 mm) in the right lateral compartment IV (Panel a). A small grade 4 FDG focus was also reported by both readers on HN PET (Panel f) in front of an apparently normal LN (short-axis: 4 mm; long axis: 8 mm) located in the left lateral compartment II (Panel d). This FDG focus was not reported on WB PET (Panel e). Node/muscle background ratio was 6.0 on HN PET vs. 3.9 on WB PET. After bilateral neck dissection, pathology confirmed HN PET/CT data.

Of the 15 patients with a true-positive PET, the mean number per patient of FDG foci rated malignant (score ≥4) was significantly higher on the HN PET than on the WB PET (2.2 vs. 1.3, P = 0.002). Of the 27 pathology-proven malignant nodes, 21 were detected on HN PET and 12 on WB PET resulting in a significantly higher detection by HN PET than by WB PET (78% [95%CI, 57–91%], vs. 44% [95%CI, 28–63%], P = 0.006). Fig 3 shows the ability of HN PET to detect more FDG-avid lesions than WB PET in the same patient.

**Fig 4:**
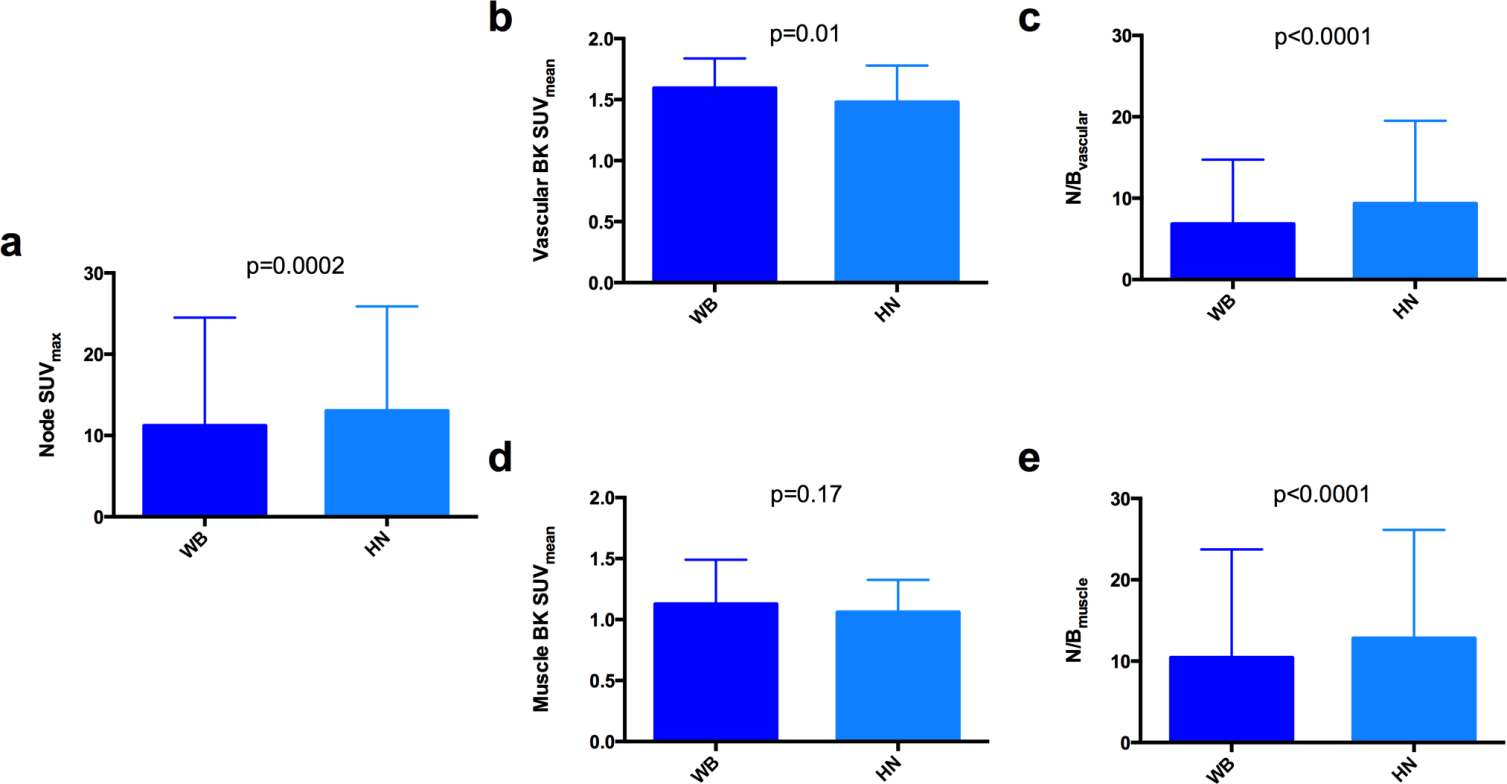
SUVmax values of the malignant lesions on head and neck (HN) and whole-body (WB) PET (Panel a), SUVmean values on HN and WB PET for vascular (Panel b) and muscle (Panel d) background, and the resulting node/background (N/B) ratios when considering either vascular (Panel c) or muscle (Panel e) background.

The N/B ratios were significantly higher on the HN than on the WB PET when considering either vascular (9.3 ± 10 vs. 6.8 ± 7.8, P<0.0001) or muscle background (12.8 ± 13.1 vs. 10.4 ± 13.1, P<0.0001) (Fig 4).

**Fig 5:**
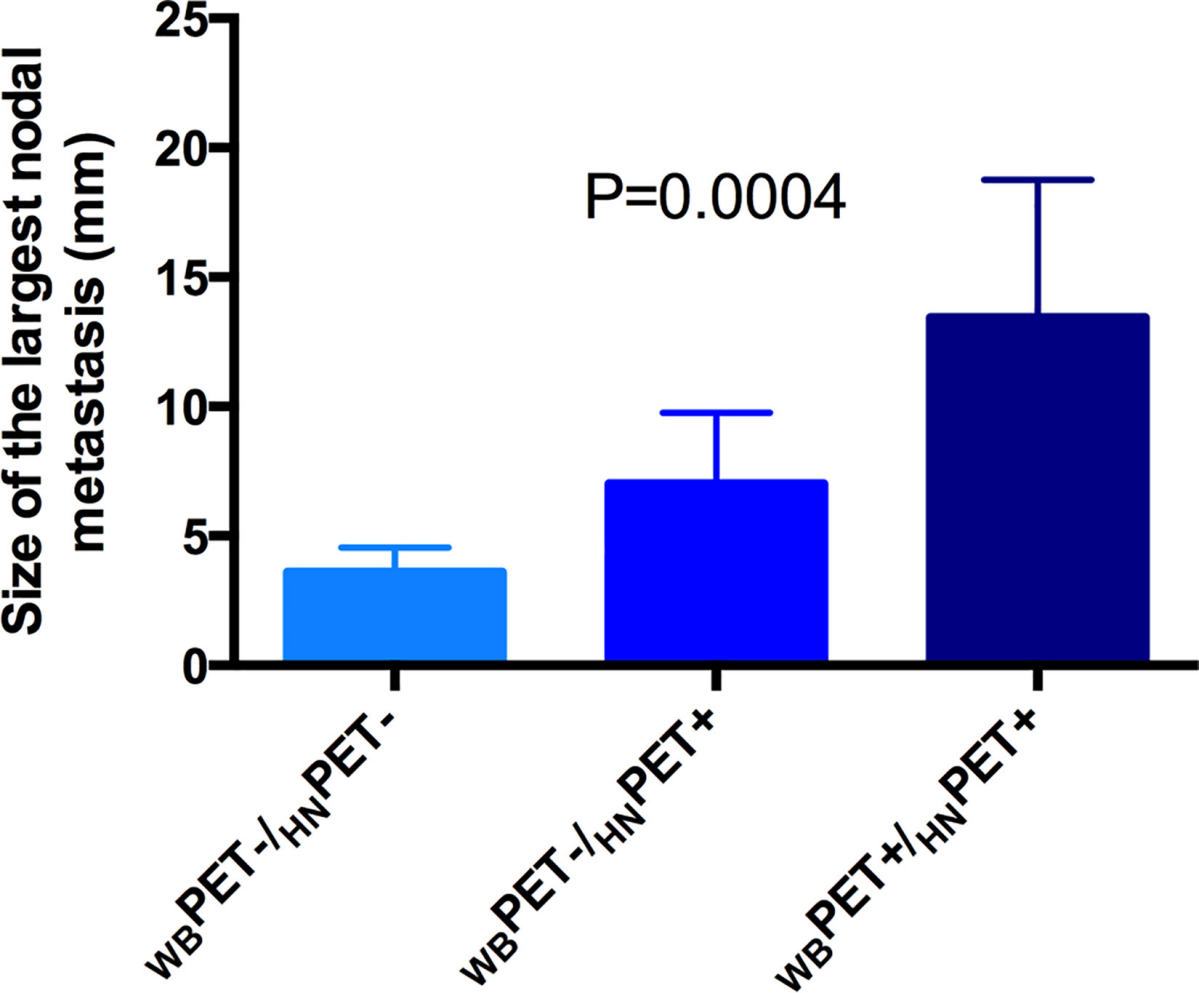
Size of the largest LN metastasis detected neither by whole-body (WB) nor head and neck (HN) PET (3 ± 1 mm), for the metastasis detected by HN but not by WB PET (7 ± 3 mm) and for those detected by both acquisitions (13 ± 5 mm) (P = 0.0004).

Overall, the mean size of the largest nodal metastasis in the 15 patients with FDG uptake was 9 ± 5 mm. This size was 3 ± 1 mm for the LN detected neither on WB nor on HN PET, 7 ± 3 mm for LN detected on HN but not on WB PET and 13 ± 5 mm for those detected on both acquisitions (P = 0.0004) (Fig 5).

The relationship between nodal tumor size, detection on WB or HN PET acquisition and FDG uptake is presented in Table 2.

**Table 2:** Metastatic lymph nodes removed during surgery: relationship between tumor size, PET detection on either WB or HN PET and FDG uptake.

Patient	Tumor Size (mm)	WB PET	SUVmax LN	SUVmean muscle BK	N/B muscle	SUVmean Vasc BK	N/B vasc	HN PET	SUVmax LN	SUVmean Muscle BK	N/B muscle	SUVmean Vasc BK	N/B vasc
6	2	No	-	1.6	-	1.8	-	No	-	1	-	1.8	-
10	3	No	-	0.6	-	1.1	-	No	-	1	-	1.1	-
6	4	No	-	1.6	-	1.8	-	No	-	1	-	1.8	-
6	4	No	-	1.6	-	1.8	-	No	-	1	-	1.8	-
10	4	No	-	0.6	-	1.1	-	No	-	1	-	1.1	-
14	4	No	-	1.2	-	1.9	-	Yes	4.5	1.1	4.1	1.8	2.5
7	5	No	-	1.3	-	1.6	-	Yes	2.8	1.2	2.3	1.5	1.9
10	5	No	-	0.6	-	1.1	-	Yes	6.6	1	6.6	1.1	6.0
10	5	No	-	0.6	-	1.1	-	Yes	2.5	1	2.5	1.1	2.3
3	5	Yes	6.3	1.8	3.5	1.9	3.3	Yes	9.9	1.5	6.6	2	4.9
6	6	No	-	1.6	-	1.8	-	Yes	7.4	1	7.4	1.8	4.1
13	6	No	-	0.8	-	1.2	-	Yes	2.4	0.9	2.7	1.3	1.8
15	7	Yes	4.5	1.6	2.8	1.8	2.5	Yes	4.3	1.9	2.3	2.4	1.8
7	7	Yes	22.3	1.3	17.1	1.6	13.9	Yes	29.9	1.2	24.9	1.5	19.9
4	8	No	-	1.4	-	1.7	-	Yes	3.1	0.8	3.9	1.5	2.1
8	9	No	-	1.2	-	1.6	-	No	-	1.2	-	1.6	-
5	12	Yes	9.6	1.9	5.0	1.7	5.6	Yes	13	1.7	7.6	1.7	7.6
8	12	Yes	9.7	1.2	8.1	1.6	6.1	Yes	11.6	1.2	9.7	1.6	7.2
10	12	Yes	5.3	0.6	8.8	1.1	4.8	Yes	8.7	1	8.7	1.1	7.9
1	13	No	-	1.1	-	1.1	-	Yes	3.6	1	3.6	1.8	2
6	15	Yes	19	1.6	11.9	1.8	10.6	Yes	25	1	25	1.8	13.9
6	17	Yes	26.8	1.6	16.7	1.8	14.9	Yes	22	1	22	1.8	12.2
13	18	Yes	5.5	0.8	6.9	1.2	4.6	Yes	9	0.9	10	1.3	6.9
5	20	Yes	20.9	1.9	11	1.7	12.3	Yes	24.6	1.7	14.5	1.7	14.5
2	21	Yes	5.7	1	5.7	1.5	3.8	Yes	4.8	1	4.8	1.7	2.8

Tumor size refers to the largest size of the lymph node (LN) metastasis; WB PET: whole-body PET; SUVmax LN: SUVmax measured in the metastatic lymph node; SUVmean muscle BK: SUVmean measured in the muscle background; N/B muscle: node/background (muscle) ratio; SUVmean vasc BK: SUVmean measured in the vascular background; N/B vasc: node/background (vascular) ratio; HN PET: head and neck PET.

#### CT scan of PET/CT

The mean dose–length product was 564 ± 148 mGy.cm for the CT of the WB acquisition and 186 ± 41 mGy.cm for the CT of the HN acquisition. The mean effective dose was 3.2 ± 0.9 mSv for the CT of the WB acquisition and 1.1 ± 0.2 mSv for the CT of the HN acquisition.

Based on the Choi classification, of the 15 patients with a positive WB or HN PET/CT study, hybrid CT scan images displayed 5 abnormal lesions (LN or abnormal mass in the thyroid bed) in 3 patients on the WB acquisition and 7 abnormal lesions in 5 patients on the HN acquisition. As a result, no CT abnormalities were reported for 79% of the FDG foci that scored 4 or 5 on the WB PET/CT and 74% on the HN PET/CT. Of the 10 patients with FDG-non-avid lesions, the hybrid CT scan showed abnormal LN in 3 patients.

### ROC analysis

**Fig 6:**
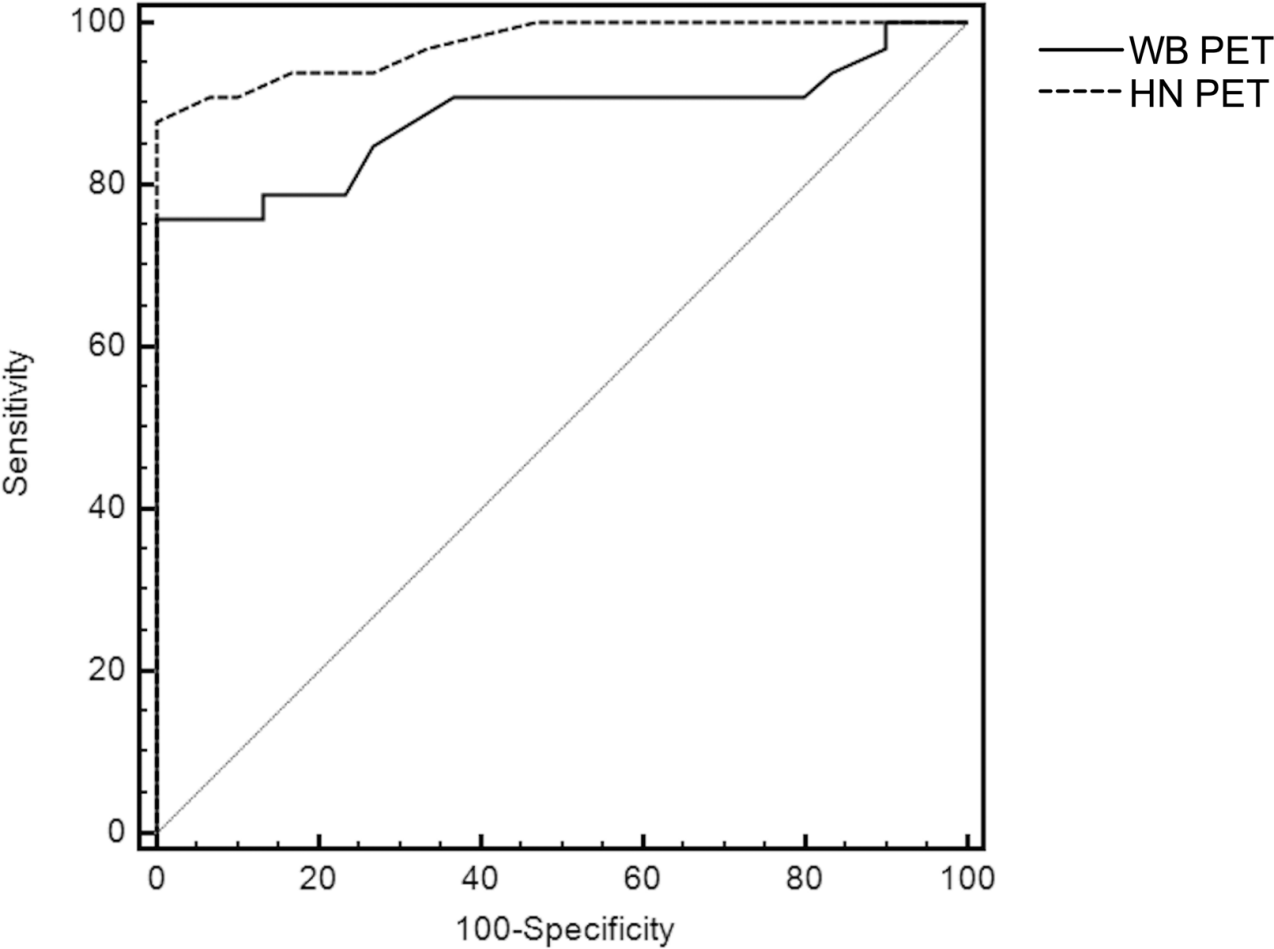
Receiver-Operating Characteristic (ROC) curve analysis for SUVmax on head and neck (HN) and whole-body (WB) PET. The area under the curve (AUC) is higher for HN PET than for WB PET (0.97 [95%CI, 0.90–0.99] vs 0.88 [95%CI, 0.78–0.95], P = 0.009).

ROC curve analysis showed that AUC was higher for HN PET than for WB PET (0.97 [95%CI, 0.90–0.99] vs 0.88 [95%CI, 0.78–0.95], P = 0.009). The optimal SUVmax threshold value was 3.1 for HN PET, leading to a 88% (95%CI, 72–97) sensitivity and to a 100% (95%CI, 88–100) specificity, while the optimal SUVmax threshold value was 3.2 for WB PET, leading to a 76% (95%CI, 58–89) sensitivity and to a 100% (95%CI, 88–100) specificity (Fig 6).

### Patient outcome

The median follow-up after surgery was 43 (2–59) months. At last follow-up visit, 10 of 26 patients showed no evidence of residual disease, seven had biochemical disease, seven had persistent disease with structural lesions and two had died by causes related to disease progression. Outcome of each 15 patients with FDG-avid lesions is reported in Table 1.

## Discussion

The present study shows that adding an HN acquisition to a WB PET/CT study highly improves the ability to evidence PRD in the neck in patients with iodine-refractory disease and serum Tg elevation. Our data demonstrated that, in comparison to WB PET, HN PET detected more FDG-avid lesions, of smaller size and with a better node/background ratio. As a result, a proportion (20%) of PET/CT studies interpreted as benign on WB PET acquisition was correctly upstaged as malignant on HN PET in the surgical group of patients with FDG-avid lesions. This suggests that a complementary HN PET is clinically relevant even when the WB PET is considered as normal at neck level.

The smallest lesion in the group of patients in whom WB PET was negative whereas HN PET was positive was 4 mm. These data are in accordance with a previous work which focused on nodal detection in the axilla in patients with breast cancer using PET/CT with PSF reconstruction [16].

The improved performance of the complementary HN acquisition is related to several factors. Firstly, the 256×256 matrix of the HN PET together with the use of PSF modelling improves the detection of small lesions in comparison to the 168×168 matrix of the WB PET. Secondly, time acquisition per bed position is longer for HN PET than for WB PET. Also, arms are positioned along the body during HN PET. Lastly, HN PET examination was always performed after the WB PET scan and thus benefited from a slightly higher uptake time, which in turn may have increased tumor uptake, as reported in studies on delayed or dual time-point PET imaging. These factors contribute to increase node/background ratios as shown in the present study. Nevertheless, the difference in tumor uptake time between HN PET and WB PET was very low and is likely to have had a minimal influence on the difference between these two PET data sets. The CT images obtained during the HN acquisition are also of higher quality compared with those acquired during the WB study. This makes the localization of FDG foci easier and enables better analysis of the possible associated tissue abnormalities. The LN located in front of FDG foci scored at 4–5 are reported as abnormal in only 26% of cases on the hybrid CT scan of the HN study, a proportion which is similar to that observed during the WB study (21%). This confirms that functional PET imaging performed better than CT without contrast media injection. This dedicated HN PET/CT does not require specific equipment and is not time-consuming. The supplemental X-ray exposure due to the hybrid CT scan of the HN acquisition is relatively low, representing about one third of that related to the CT scan corresponding to the WB acquisition. Hence, we believe that an HN acquisition can be routinely performed after WB acquisition in all the patients for whom a PET/CT study is aimed to evidence PRD in the neck.

Until now, only a few studies have focused on the neck with a dedicated acquisition in DTC patients [12–14]. Davison et al. have reported that the HN PET increased the number of FDG foci scored as malignant. However, the number of patients was limited and no comparison with pathology was presented in this study [12]. As recently reviewed [23], delayed time-point imaging has been shown to be relevant in some malignancies by increasing FDG uptake in tumor cells and clearance of background activity. Delayed imaging with several neck PET acquisitions was recently assessed to differentiate metastatic thyroid cancer from benign uptake in sub-centimetre cervical LN [13]. A limitation of the protocol of Kunawudhi et al. is to markedly increase the time duration of PET/CT. Also, no comparative data between WB and HN PET were available. It was recently reported that an HN PET improves readers’ performance [14]. But no comparison was performed between PET results and pathology.

Comparison between PET/CT and neck US was not possible in our retrospective study, especially because US was not performed in all patients; however, both imaging modalities are useful and complement each other, even if US is of limited value in central neck [22,24]. Although uncommon, some malignant lesions in DTC accumulate neither ^131^I nor ^18^F-FDG [25]. In this situation, US is very useful to identify suspicious LN and to guide fine-needle aspiration biopsy for cytology and for Tg measurement in the aspirate fluid [26]. In the present series, 10 patients with FDG-non avid lesions were operated on mostly according to US data. In patients with FDG-avid lesions, PET/CT can rule-out distant metastases and HN acquisition offers complementary relevant data to the surgeon. In our series, HN PET provided more information than neck US to detect LN disease in 8 out of 11 patients. In one patient, only HN PET suggested the presence of contralateral LN involvement. These data enable the surgeon to more accurately plan the procedure of the LN dissection re-intervention. Therefore, it could be recommended to perform systematically HN PET/CT in addition to neck US in all the iodine-negative patients before neck surgery.

Some studies have reported limited sensitivity of PET when serum Tg levels are below 10 ng/ml [27] leading to recommend this imaging modality in patients with a Tg level above this limit [28,29]. In our study, 8 out of the 15 patients with FDG-avid tumor lesions had a serum Tg level under levothyroxine ≤10 ng/ml. Interestingly, two out of the three patients with false-negative WB PET and true-positive HN PET data displayed a Tg level ≤10 ng/ml, and all three patients either achieved cure after surgery or showed biochemical disease at the last follow-up visit. These data strongly suggest that PET/CT can be indicated below this Tg cut-off of 10 ng/ml and that an early detection of limited LN lesions on PET/CT can lead to early surgical management and favourable outcome. Additionally, as previously reported [30], PET/CT was effective to depict PRD even without rhTSH stimulation.

A concern might be to increase the number of indeterminate foci on HN PET in comparison to WB PET. Such dubious findings were not increased in the surgical group. Moreover, WB and HN PET are read together in clinical practice. We also reported excellent inter-observer agreement for reading. Interestingly, no false-positive lesion was reported either on WB or HN PET, suggesting that lesions scoring 4–5 can be interpreted with confidence as truly malignant.

Lastly, it should be noted that images in the present study were reconstructed with PSF reconstruction, an algorithm that improves spatial resolution throughout the entire field of view and allows detection of smaller cancer deposits [16,17]. Other major software technologies have been released, such as time of flight, which improves PET imaging in overweight patients [31], and more recently Bayesian penalised likelihood reconstruction, which allows an increased number of iterations without the increased noise usually seen in conventional OSEM reconstructions [32]. These technologies can be combined with PSF modelling and could further improve the diagnostic performance of PET for the detection of PRD at the cervical level.

## Conclusion

HN acquisition, when performed with a longer acquisition time per bed position and a thinner matrix, significantly improves PET/CT performances for the detection of PRD at the cervical level compared with the WB acquisition alone. We recommend systematically adding an HN acquisition when PET/CT is performed in this setting, as this method is not time-consuming and can be planned in advance, thus not perturbing the time schedule of a busy PET unit.
